# Characterization of peripartum cardiomyopathy by cardiovascular magnetic resonance imaging

**DOI:** 10.1186/1532-429X-17-S1-Q46

**Published:** 2015-02-03

**Authors:** Arash Haghikia, Philipp Röntgen, Jens Vogel-Claussen, Denise Hilfiker-Kleiner, Johann Bauersachs

**Affiliations:** 1 Cardiology and Angiology, Hannover Medical School, Hannover, Germany; 2 Radiology, Hannover Medical School, Hannover, Germany

## Background

Peripartum Cardiomyopathy (PPCM) is a potentially life threatening disease and the major cause of acute heart failure in the peripartum period. While many aspects of its clinical profiles have been frequently reported, functional analysis, in particular of the right ventricle, and tissue characterization by cardiovascular magnetic resonance (CMR) imaging have been only sporadically described. The aim of the present study was to analyze pathological alterations found in CMR imaging of patients newly diagnosed with PPCM.

## Methods

This was a multicenter study enrolling 34 patients with confirmed PPCM who underwent CMR imaging at the time of diagnosis.

## Results

Cine imaging of PPCM patients showed moderate to severe reduction of systolic left ventricular (LV) function (mean LVEF: 29.7±12.8%). In 35% of the patients right ventricular (RV) systolic function was also reduced with a mean RVEF of 42.9±13.9%. Dilatation of the LV was observed in 91% (mean LV-EDV/BSA 128.5±32.1 ml/m^2^) and dilatation of the RV was present in 24% (mean RV-EDV/BSA 87.4±18.5 ml/m^2^) of the patients. Focal non-ischemic late gadolinium enhancement (LGE) was visible in 71% and regional wall motion abnormalities were evident in 88% of the patients. LGE and wall motion abnormalities were predominantly located in the anteroseptal and basal to midventricular segments.

## Conclusions

Beside LV systolic dysfunction, RV dysfunction and dilatation are frequently observed in PPCM patients at the time of diagnosis. The presence of LGE suggests focal damage of myocardial tissue. A distinct pattern of LV wall motion abnormalities is evident in most PPCM patients. The present study may help to establish a set of CMR criteria suitable for diagnosis in patients with suspected PPCM and contribute to our understanding of this diesease.

## Funding

This study was supported by the German Research Foundation (DFG), the German Federal Ministry of Education and Research (BMBF) and the Cluster of Excellence "Regenerative Biology to Recontructive Therapy" (Rebirth).

